# Clinical Evidence of the Efficacy of a Mouthwash Containing Propolis for the Control of Plaque and Gingivitis: A Phase II Study

**DOI:** 10.1155/2011/750249

**Published:** 2011-03-31

**Authors:** Elizete Maria Rita Pereira, João Luís Duval Cândido da Silva, Fernando Freitas Silva, Mariana Passos De Luca, Efigênia Ferreira e Ferreira, Telma Campos Medeiros Lorentz, Vagner Rodrigues Santos

**Affiliations:** ^1^Laboratory of Microbiology and Biomaterials, Department of Oral Clinical, Oral Pathology, Oral Surgery, Faculty of Dentistry, Federal University of Minas Gerais, Campus Pampulha, Avenida Presidente Antônio Carlos 6627, 31.270-901 Belo Horizonte, MG, Brazil; ^2^Department of Public Health, Faculty of Dentistry, Federal University of Minas Gerais, Campus Pampulha, Avenida Presidente Antônio Carlos 6627, 31.270-901 Belo Horizonte, MG, Brazil

## Abstract

The aim of this study was to evidence the clinical efficacy of an alcohol-free mouthwash containing 5.0% (W/V) Brazilian green propolis (MGP 5%) for the control of plaque and gingivitis. Twenty five subjects, men and women aging between 18 and 60 years old (35 ± 9), were included in a clinical trials phase II study who had a minimum of 20 sound natural teeth, a mean plaque index of at least 1.5 (PI), and a mean gingival index of at least 1.0 (GI). They were instructed to rinse with 10 mL of mouthwash test for 1 minute, immediately after brushing in the morning and at night. After 45 and 90 days using mouthwash, the results showed a significant reduction in plaque and in gingival index when compared to samples obtained in baseline. These reductions were at 24% and 40%, respectively (*P* < .5). There were no important side effects in soft and hard tissues of the mouth. In this study, the MGP 5% showed evidence of its efficacy in reducing PI and GI. However, it is necessary to perform a clinical trial, double-blind, randomized to validate such effectiveness.

## 1. Introduction

The first reference to mouth rinse as a formal practice is credited to Chinese medicine, about 2700 B.C.E., to treat the diseases of the gums [2]. The Gram-positive and Gram-negative bacteria that compose oral biofilms produce many metabolites that induce gingival inflammation (i.e, gingivitis). Gingivitis can lead to periodontitis, a condition in which gingival and bone tissues are destroyed. The majority of the population may not perform mechanical plaque removal sufficiently. Thus, antimicrobial mouth rinses that improve daily home care may provide an effective way of removing or controlling bacterial plaque to limit gingivitis and periodontitis [3]. The main indications are either the improvement of dental health (plaque and gingivitis elimination in particular) or the prevention of infections caused by bacteria of the oral cavity in specific situations, such as tooth extraction, intraoral surgical procedures, or immune suppression due to cancer therapy or transplantation. The use of antimicrobial mouth rinses has been proposed to reduce the levels of oral bacteria, especially *Streptococcus mutans* [4]. In fact, it has been shown that chemotherapeutic mouth rinses are an effective adjunct to regular brushing and flossing for patients with gingivitis, providing a clinically significant benefit in the reduction of plaque and gingivitis [3]. Propolis is a resinous substance collected by honeybees from buds and exudates of certain trees and plants and stored inside their hives. It has been used in folk medicine from ancient times to treat various ailments [5]. The action against microorganisms is an essential characteristic of propolis, and humans have used it for centuries for its pharmaceutical properties [6]. The antibacterial activity of propolis is reported due to flavonoids, aromatic acids, and esters present in resins. Galangin, pinocembrin, and pinostrobin are known as the most effective flavonoids agents against bacteria. Ferulic acid and caffeic acid also contribute to the bactericidal action of propolis [7]. 

The American Dental Association (ADA) Guidelines (Council on Scientific Affairs) are applied to products used for the control of gingivitis and, if applicable, supragingival dental plaque, through the use of chemotherapeutic agents. Products that control gingivitis solely by the mechanical removal of plaque are not considered by these guidelines. This council believes, that, because plaque is the etiologic agent for gingivitis and other oral diseases, the only accepted chemotherapeutic products that will be allowed to make plaque control or plaque modification claims will be those that can also demonstrate a significant effect against gingivitis. If a product can only demonstrate a significant plaque reduction without a concomitant significant reduction in gingivitis, it will not be eligible for acceptance [8]. Thus, the purpose of this study was to show the clinical evidence, in subjects, of an alcohol-free mouthwash containing Brazilian green propolis for the control of plaque and gingivitis for three months.

## 2. Material and Methods

### 2.1. Design Study and Product Tested

This was an interventional study of phase II for three months, follow-up type, and was conducted at the Faculty of Dentistry of Federal University of Minas Gerais, Brazil, from August 2009 to April 2010. The alcohol-free mouthwash containing 5% w/v of Brazilian green propolis (MGP 5%) used in that study was handled according to our request by Pharma Néctar (Belo Horizonte), within the standards required by ANVISA [9] and within the requirements of ISO 9001 and GMP International. Five percent of propolis dry extract (w/v) was added to a solution containing glycerin, sodium benzoate, and purified water.  Table 1 shows the amount in mg/g, the main chemical markers found in green propolis used in the formulation of the mouthwash study.

### 2.2. Participants

Twenty five subjects, of age varying from 18 to 60 years (median age 35 ± 9), with generally good health, not pregnant nor breastfeeding, who met the following inclusion criteria, were included into the study: a minimum of 20 sound, natural teeth; a mean plaque index (PI) [10] of at least 1.5; a mean gingival index (GI) [11] of at least 1.0. Subjects with orthodontic appliances or removable prosthetics, tumors of the soft or hard oral tissues, and advanced periodontal disease, receiving antibiotic therapy 2 weeks before the beginning of the study, or presenting hypersensibility to propolis were excluded. Third molars and those teeth with cervical restorations or prosthetic crowns were not included in the tooth count. The selection of participants was made by convenience, based especially on the availability to the study, while the study was conducted. All subjects read and signed informed consent forms before the start of the study. The protocol for the study was approved by the local ethical review committee—Committee of Bioethics in Research at the Federal University of Minas Gerais (COEP/UFMG-0600/09), and registered in Clinicaltrials.gov (NCT01142843).

### 2.3. Assessing Mouthwash

The examination baseline consisted of a complete soft and hard tissues examination that was performed to register the condition of oral mucosa, so that any changes in the course of the study could be identified, making assessment to whether these changes could be related to the mouthwash. The gingivitis of the mesiobuccal, midbuccal, distobuccal, mesiolingual, midlingual, and distolingual of all eligible teeth was scored using the Talbott modification Gingival Index of the Löe-Silness [11], in which the gum was scored on a four-point scale from 0 (absence of inflammation) to 3 (severe inflammation). The supragingival plaque of the mesiobuccal, midbuccal, distobuccal, mesiolingual, midlingual, and distolingual of all eligible teeth was scored using the Turesky modification of the Quigley-Hein Plaque Index [10]. Previously, disclosing with erythrosine 3% solution, plaque was scored on a six-point scale from 0 (no plaque) to 5 (plaque covers two-thirds or more of the tooth surface). Each tooth was divided into six areas, three buccal (mesiobuccal, midbuccal, distobuccal) and three lingual (mesiolingual, midlingual, distolingual), and the plaque quantified using the Turesky modification of the Quigley–Hein Plaque Index [10]. There were also evaluated Severity Plaque Index and Severity Gingival Index [12, 13]. These index measured the rate of the surface that had high count of plaque (count similar to 3, 4, 5 of the modification Quigley-Hein Plaque Index [10] and high gingival index (count similar to 2, 3 modification Gingival Index of the Löe-Silness [11]. These examinations were all repeated after 45 and 90 days of the use of the mouthwash. After baseline examination, each subject received a complete oral prophylaxis, which included the removal of all supragingival plaque and calculus deposits. Soon after, the subjects received an alcohol-free mouthwash containing 5% green propolis (MGP 5%) and a toothbrush. They were instructed to brush their teeth as usual and to rinse with the 10 mL of MGP 5%, twice a day, for one minute, right after their meals in the morning and at night. Participants were required not to use another mouthwash throughout the study. When new supplies were issued, subjects returned their used materials, so that the compliance to the product could be monitored.

### 2.4. Reproducibility of Clinical Examinations

All examinations were conducted by a single examiner trained to optimize the consistency of the study. Prior to the study, the adviser trained the dental examiner, as a “gold standard,” directing him to introduce the periodontal probe, gently, into the gingival sulcus, keeping the instrument parallel to the long axis of the tooth, sliding it from the distal to the mesial so delicately in the buccal and lingual surface of each evaluated tooth. For calibration, there were examined nine subjects not included in the study. For the plaque index it was performed a theoretical calibration. Soon after, photos were used to obtain a standardization intraexaminer. The photos were exhibited by the adviser to the examiner that noted the values of plaque index corresponding to each picture. After 15 days, the same pictures were exposed to the examiner that registered again the values of the plaque index. Next, the plaque index obtained in the first and second time was compared to verify the level of intraexaminer. Then, we obtained a kappa value of 0.73, considered a substantial estimate of reliability [14].

### 2.5. Data Analysis

The statistical package BioEstat version 4.0 was used for data analysis in this study. The average adjusted in the baseline for both scores of the modified Plaque Index of Quigley-Hein [10] and modified Gingival Index of Löe-Silness [11] as for the corresponding severity scores, for being a nonparametric distribution, was compared through covariance analysis, by Friedman test for data obtained at 45 and 90 days of the study. All statistical tests of hypotheses had two strands and a significance level of *P* < .05 was considered [15, 16].

## 3. Results

**Figure 1:**
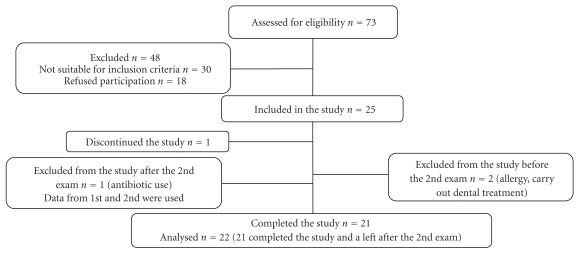
Flow diagram about desgining of study.

During the period available for the development of the study was carried out a selection for convenience in which 73 patients were eligible. Because of the inclusion and exclusion criteria and availability, it was only possible to include a sample of 25 individuals, being in agreement to what was proposed [17, 18], for phase II trials (Figure 1). Twenty-one (10 males and 11 females) subjects completed the period of study. The others left the study for many reasons: one searched for dental services during the study and was excluded; another had possible allergy to propolis; one subject left the study because he felt hypersensibility on teeth, and one individual was excluded for taking antibiotics due to illness. Data for this subject are included in the 45-day analysis. 

### 3.1. Gingivitis and Gingival Severity

The mean baseline scores of Gingival Index GI, 45 and 90 days, were recorded. The MGP 5% demonstrated a reduction in gingivitis over than 40%, being statistically significant, comparing to the scores of 45 and 90 days in the baseline scores (*P* < .05). Although, comparing to the scores of 90 days to 45 days, there was not a statistically significant reduction in gingivitis (Table 2). The MGP 5% showed a reduction in rate of the surface with scores 3, 4, and 5 of plaque index. This reduction was statistically significant, (over 70%), comparing the mean baseline scores with 45 and 90 days (*P* < .05). There was not any statistical significance comparing the mean scores of 45 and 90 days (Table 3).

### 3.2. Plaque and Severity of Plaque

The mean baseline scores of plaque index (PI), 45 and 90 days, were obtained. Analysis of variance using the Friedman test was performed with the baseline scores as covariates, showing that the MGP 5% had an effect on the plaque in the examinations at 45 and 90 days, being statistically significant (*P* < .05), with a reduction of 26% and 24%, respectively. However, it did not obtain significant effect on plaque, comparing to the scores of tests performed on 45 and 90 days (Table 4). In Severity Plaque Index was observed a statistically significant difference comparing to the mean baseline scores with 45 and 90 days. There was a reduction of 41% in the second exam, remaining the same after the third exam (*P* < .05). This significant difference was not observed in exams performed on 45 and 90 days (Table 5).

### 3.3. Oral Mucosa Changes

In the last oral examination conducted by the researcher was observed, only in one subject, the presence of an exophytic lesion, located in the free marginal gum of the labial surface of the element 27. This lesion had a reddish surface, smooth, bleeding to touch, measuring 2 × 2 mm. Plaque accumulation was observed around the lesion. Given these facts, it was suggested a possible diagnosis of pyogenic granuloma. After that, it was made a supra- and subgingival scaling in this lesion area, resulting in its complete regression in a period of 15 days. One patient reported a burning feeling in the oral mucosa for a short period of time every time he used the mouth rinse during the three months of treatment. Another 3 patients reported that during the period using mouth rinse they had a dryness sensation in the mouth.

## 4. Discussion

This phase II trial study evaluated the action of MGP 5% on gingivitis and periodontopathogenic plaque. 

Clinical trials often present limitations independent on the efforts of the researchers. This study has limitations as the presence of unexpected reactions to the product and as a probable allergic reaction, that did not deserve any concern, but resulted in the exclusion of an individual and decreased the sample. Another limitation was the difficulty to control the compliance to the study, how to get in touch with them every time they needed to return for evaluation. Despite the imposition of a control-use mouthwash (return the empty bottle), clinical trials have limitations with respect to the veracity of the suitability of the product by patients that are generally beyond the control of the researcher. The MGP 5% produced significant reductions in supragingival plaque and gingivitis as adjunct to the oral hygiene procedures, when compared to baseline scores index with 45 and 90 days. These findings are probably justified by the antibacterial and anti-inflammatory effects of propolis. The reduction of number of microorganisms in dental plaque resulted in decreasing of bulk. There are some studies *in vitro *and *in vivo *where propolis, in several formulations, has demonstrated activity against periodontal pathogens [19–21]. The antimicrobial property of Brazilian propolis is attributed to the presence of flavonoids, phenolic acids, and their prenylated derivatives on its composition. Propolis has a complex chemical composition, considering the type of bee that produced it, the origin, and seasons of collection. Moreover, its action is dose-time dependent, and, in this study, we took into account the time of use, evaluation times, and the concentration of the mouth rinse. Some components present in propolis as flavonoids (quercetin, galangin, and pinocembrin), caffeic acid, benzoic acid, and cinnamic acid, probably act on the microbial membrane or surface of the cell wall, causing structural and functional damage [22]. Synergistic effects of different compounds seem to be the most important process to explain the antibacterial activity of propolis, since it is well established that a single propolis component does not have an activity greater than the other components of propolis isolated [19]. This study used the green propolis, derived from *Baccharis dracunculifolia*, a native plant of southeastern Brazil [23]. This kind of propolis has as its main bioactive compounds the artepillin C and others compounds as coumarinic acids that are probably related to anti-inflammatory and antibacterial properties, respectively. The artepillin C, in other studies, showed potential anti-inflammatory activity [24–26]. Other components presented in green propolis might be involved in anti-inflammatory effects observed on its results. Propolis, to produce the anti-inflammatory effect, acts in the modulation of cytokines and inflammatory enzymes, such as the suppression of the production of prostaglandins, leukotrienes, histamine, and TGF-*β*1 [26, 27]. Reducing the number of microorganisms in dental plaque results in the reduction of products released by them, which act as trigger of gingival inflammation. The results of MGP 5% on the severity of plaque and gingival indexes suggest that the anti-inflammatory effect on the gingival condition of subjects was greater than the effect of reducing plaque, due to the higher decrease of bleeding points than the decrease of plaque. 

The treatment period of 3 months was chosen because of gingivitis is a chronic disease and because there was no interference in the habits of hygiene of the individuals involved in the study. In this study, we applied an approach close to the required standards by the ADA for testing new products for use in the oral cavity. The ADA general purpose for such products and methods is to assist the identification of sites or subjects with existing periodontitis or at increased risk of periodontitis, or the development or progression of periodontitis. Clinical use of such diagnostics might occur (1) during initial evaluation (screening, pretreatment risk assessment, diagnosis, and treatment planning); (2) during treatment or management (monitoring therapeutic endpoints and identifying therapeutic targets), and (3) posttreatment (establishment of recall intervals and early detection of recurrent disease). The specifics of clinical trial design for each of these related, but separate, clinical functions may differ depending on a variety of circumstances [28]. 

As a phase II study, there is not a crucial requirement in relation to time of study [29, 30]. The concentration of 5% was chosen considering that propolis is a resinous substance that, at higher concentrations, could cause its precipitation in the bottle, staining the teeth and, as a result, not being accepted by patients due to the strong flavor. So far, there are not any clinical studies that evaluated the oral rinse of propolis on the basis of plaque and gingivitis control for such a long period, as we did [29, 31, 32]. During the three months of study, oral examinations were performed in recalls after the baseline examination, and one patient had a pyogenic granuloma in the buccal marginal gum of the element 27. This probably occurred because the patient did not have an appropriate oral hygiene, as verified by oral examination, according to the presence of plaque accumulation in this tooth. The plaque removal by scaling, supra- and subgingival plaque, and the regression of the lesion after 15 days support this hypothesis [33]. Those subjects, who reported dryness mouth and rough, probably had that feeling because the ingestion of water was not enough to body hydration. Moreover, these patients were female and were in the age group corresponding to the period of menopause. When the subject started to drink the required amount of water, the feeling disappeared. Although the MGP 5% is alcohol-free, there was one patient who reported a burning feeling when using the mouth rinse for three months. Probably, this fact was due to the concentration of the mouthwash being 5%, which has a strong flavor, while the other participants have not reported the same. In intraoral examination, this patient had no mucosal irritation. 

More recently, for various reasons, there has been an increase in the demand for alcohol-free mouthwashes. The reason for not including alcohol in the mouthwash was initially based on both social and health reasons. Social reasons include religious objections and the potential for detecting alcohol in the breath. Additionally, it has been recognized that some individuals' oral mucosa is sensitive to alcohol, with some evidence to show that discomfort increases linearly with increasing concentrations of alcohol. Other potential problems with alcohol rinses include the softening and reduced color stability of tooth-colored restorations and the possible increased risk of developing oral cancer [34].

## 5. Conclusions

The present study showed evidence of the efficacy of alcohol-free mouthwash containing 5% of Brazilian green propolis for the control of plaque and gingivitis, suggesting that it can be used as a therapeutic and preventive use for the control of periodontal diseases. However, it is necessary to perform a clinical trial, double-blind, randomized according to the requirements of the Council on Dental Therapeutics of the American Dental Association (ADA) to prove the efficacy of an alcohol-free mouthwash containing 5% w/v of Brazilian green propolis for the use of this product to become customary in dentistry.

## Figures and Tables

**Table 1:** Chemical constituents identified and quantified (markers) by reverse-phase high-performance liquid chromatography: flavonoids and other chemical constituent present in a gram of a Brazilian green propolis sample used in handling (SBN 97) [1].

No.	COMPOUNDS	UNIT (mg/g)	RESULTS
1	CUMARINIC ACID	mg/g	3.56
2	CINNAMIC ACID	mg/g	1.66
3	QUERCENTIN	mg/g	1.38
4	KAEMPFEROL	mg/g	1.77
5	ISORHAMNETIN	mg/g	0.91
6	SAKURANETIN	mg/g	5.57
7	PINOBANSKIN-3-ACETATE	mg/g	13.92
8	CHRYSIN	mg/g	3.51
9	GALANGIN	mg/g	9.75
10	KAEMPFERIDE	mg/g	11.60
11	ARTEPILLIN c	mg/g	82.96
	(3,5-DIPRENYL-4-HIDROXYCINNAMIC ACID)		

**Table 2:** Mean scores of Gingival Index (DP) and percent reduction between periods.

	Baseline	45 days	90 days	Reduction-%		
MGP 5%	*n* = 22	*n* = 22	*n* = 21	Baseline–45 days	Baseline–90 days	45 days–90 days
	1.17 (0.20)	0.64 (0.24)	0.70 (0.18)	45*	40*	—

*Friedman test (ANOVA) *P* < .05.

**Table 3:** Mean scores of Severity Gengival Index (DP) and percent reduction between periods.

	Baseline	45 days	90 days	Reduction-%		

MGP 5%	*n* = 22	*n* = 22	*n* = 21	Baseline–45 days	Baseline–90 days	45 days–90 days
	0.30 (0.17)	0.08 (0.06)	0.07 (0.03)	73*	77*	13 (ns)**

*Friedman test (ANOVA) *P* < .05.

**Not significant.

**Table 4:** Mean scores of Plaque Index (DP) and percent reduction between periods.

	Baseline	45 days	90 days	Reduction-%		
MGP 5%	*n* = 22	*n* = 22	*n* = 21	Baseline–45 days	Baseline–90 days	45 days–90 days
	2.39 (0.69)	1.77 (0.61)	1.82 (0.62)	26*	24*	—

*Friedman test (ANOVA) *P* < .05.

**Table 5:** Mean scores of Severity Plaque Index (DP) and percent between periods.

	Baseline	45 days	90 days	Reduction-%		
MGP 5%	*n* = 22	*n* = 22	*n* = 21	Baseline–45 days	Baseline–90 days	45 days–90 days
	0.44 (0.19)	0.26 (0.14)	0.26 (0.15)	41*	41*	—

*Friedman test (ANOVA) *P* < .05.
